# *N*-Glycosylation of cholera toxin B subunit in *Nicotiana benthamiana*: impacts on host stress response, production yield and vaccine potential

**DOI:** 10.1038/srep08003

**Published:** 2015-01-23

**Authors:** Krystal Teasley Hamorsky, J. Calvin Kouokam, Jessica M. Jurkiewicz, Bailey Nelson, Lauren J. Moore, Adam S. Husk, Hiroyuki Kajiura, Kazuhito Fujiyama, Nobuyuki Matoba

**Affiliations:** 1 Owensboro Cancer Research Program of James Graham Brown Cancer Center at University of Louisville School of Medicine, Owensboro, KY, USA; 2 Department of Medicine, University of Louisville School of Medicine, Louisville, KY, USA; 3 Department of Pharmacology and Toxicology, University of Louisville School of Medicine, Louisville, KY, USA; 4 The International Center for Biotechnology, Osaka University, Osaka, Japan

## Abstract

Plant-based transient overexpression systems enable rapid and scalable production of subunit vaccines. Previously, we have shown that cholera toxin B subunit (CTB), an oral cholera vaccine antigen, is *N*-glycosylated upon expression in transgenic *Nicotiana benthamiana*. Here, we found that overexpression of aglycosylated CTB by agroinfiltration of a tobamoviral vector causes massive tissue necrosis and poor accumulation unless retained in the endoplasmic reticulum (ER). However, the re-introduction of *N*-glycosylation to its original or an alternative site significantly relieved the necrosis and provided a high CTB yield without ER retention. Quantitative gene expression analysis of *PDI*, *BiP*, *bZIP60*, *SKP1*, *26Sα* proteasome and *PR1a*, and the detection of ubiquitinated CTB polypeptides revealed that *N*-glycosylation significantly relieved ER stress and hypersensitive response, and facilitated the folding/assembly of CTB. The glycosylated CTB (gCTB) was characterized for potential vaccine use. Glycan profiling revealed that gCTB contained approximately 38% plant-specific glycans. gCTB retained nanomolar affinity to GM1-ganglioside with only marginal reduction of physicochemical stability and induced an anti-cholera holotoxin antibody response comparable to native CTB in a mouse oral immunization study. These findings demonstrated gCTB's potential as an oral immunogen and point to a potential role of *N*-glycosylation in increasing recombinant protein yields in plants.

Plants may be a viable alternative to producing complex recombinant proteins over conventional cell culture-based systems with the potential for providing both cost effectiveness and scalability^1^,^2^,^3^, in addition to a low risk for human pathogen contamination^4^,^5^. The recent FDA marketing approval of the first plant-made biopharmaceutical for human use, a recombinant form of glucocerebrosidase produced in carrot suspension cells^6^,^7^ further validates plants as powerful production factories for recombinant pharmaceutical proteins.

An ultimate goal of plant-made biopharmaceuticals research is to produce proteins at high levels. This is often achieved by targeting proteins through the secretory pathway^8^,^9^, given that the endoplasmic reticulum (ER) lumen houses molecular chaperones and enzymes that support proper folding and assembly of nascent polypeptides. The ER also provides the correct physiological molecular environment for disulfide bond formation and glycosylation^10^,^11^,^12^. Therefore, ER-targeting is required for the production of complex glycoproteins such as immunoglobulin (Ig)G monoclonal antibodies. The ER quality control (ERQC) system monitors proteins for proper folding and identifies improperly folded proteins for ER associated degradation (ERAD)^13^. However, heavy protein secretion or adverse environmental conditions result in accumulation of unfolded proteins in the ER, causing ER stress. Consequently, the unfolded protein response (UPR) induces up-regulation of stress response genes for protein folding.

Until recently, little was known about ER stress, UPR and ERAD in plants. Recent reviews summarized two UPR arms in plants: one induces activation of two membrane-associated transcription factors (basic-region leucine zipper [bZIP]17 and bZIP28) and the other involves the RNA-splicing factor IRE1 and its target RNA (bZIP60)^13^,^14^. Both arms activate ER stress responsive genes encoding various molecular chaperones, including luminal binding protein (BiP), calnexin, calreticulin and protein disulfide isomerase (PDI). Unfolded proteins not repaired by UPR are eliminated by ERAD in four steps: recognition, ubiquitination, dislocation and degradation by the 26S proteasome^13^. In the field of plant-made biopharmaceuticals research, it has been empirically determined that some ER-targeted recombinant proteins cause stress in the host plant, resulting in poor accumulation. In spite of this perceived “Achilles' heel” in plant-made biopharmaceuticals development, only a few studies have addressed this issue in detail in transgenic plants of non-*Nicotiana* species^8^,^15^.

Among different plant species used for recombinant protein production, *Nicotiana benthamiana* has become one of the most widely used hosts due to its capacity to support various transient overexpression systems based on viral and non-viral vectors^1^,^16^,^17^. These systems have the capacity to produce large amounts of biopharmaceuticals faster than any other expression system^17^,^18^, sometimes accumulating over 1 g of target proteins per kg of leaf material within days. It is conceivable that such intense foreign protein synthesis enforced by these vectors would trigger a significant increase in payload to the ERQC system in the host plant. Therefore, understanding ER stress fueled by transient protein overexpression in *N. benthamiana* could be of significant importance in plant-made biopharmaceuticals production systems.

In this report, we studied the production of a potent oral immunogen, cholera toxin B subunit (CTB) using *Agrobacterium*-mediated delivery of a tobamovirus replicon vector (the magnICON system) in *N. benthamiana*. One of our primary objectives was to investigate the role of *N*-glycosylation in ER stress upon transient CTB production in *N. benthamiana*. Other reports on CTB production in transgenic plants such as potato^19^, rice^20^,^21^, tomato^22^ and tobacco^23^,^24^,^25^, have addressed this issue with no or limited details. Previously, we reported that recombinant CTB expressed in transgenic *N. benthamiana* is *N*-glycosylated at Asn4^26^,^27^. We engineered an aglycosylated variant of CTB with the “KDEL”^28^ ER retention signal (termed N4S-CTB-KDEL) and overexpressed the protein in *N. benthamiana* using the magnICON system. While N4S-CTB-KDEL accumulated at a high level and retained molecular integrity and oral immunogenicity^26^, we subsequently discovered that a N4S-CTB-KDEL variant devoid of the ER retention signal (N4S-CTB) showed a notably low yield and induced severe necrosis in leaf tissue. Meanwhile, the original Asn4 *N*-glycosylated, non-ER-retained CTB (gCTB) did not cause such tissue damage and provided an exceptionally high yield, significantly greater than that of N4S-CTB-KDEL. These intriguing findings prompted us to investigate various stress markers in *N. benthamiana* overexpressing gCTB and N4S-CTB. Given the outstanding producibility, we purified and characterized gCTB using biochemical, biophysical and immunological experimentation towards possible vaccine development. These studies suggest gCTB as a potential alternative to the bacterial CTB used in an internationally licensed oral cholera vaccine. Moreover, the data reported herein contribute to our understanding of the stress response caused by transient overproduction of foreign proteins in *N. benthamiana*, and also indicate potential utilities of *N*-glycosylation for the development of plant-made glycoprotein pharmaceuticals.

## Results

### Expression of a non-ER-retained aglycosylated CTB variant by a tobamoviral vector causes severe tissue damage in N. benthamiana

**Figure 1:**
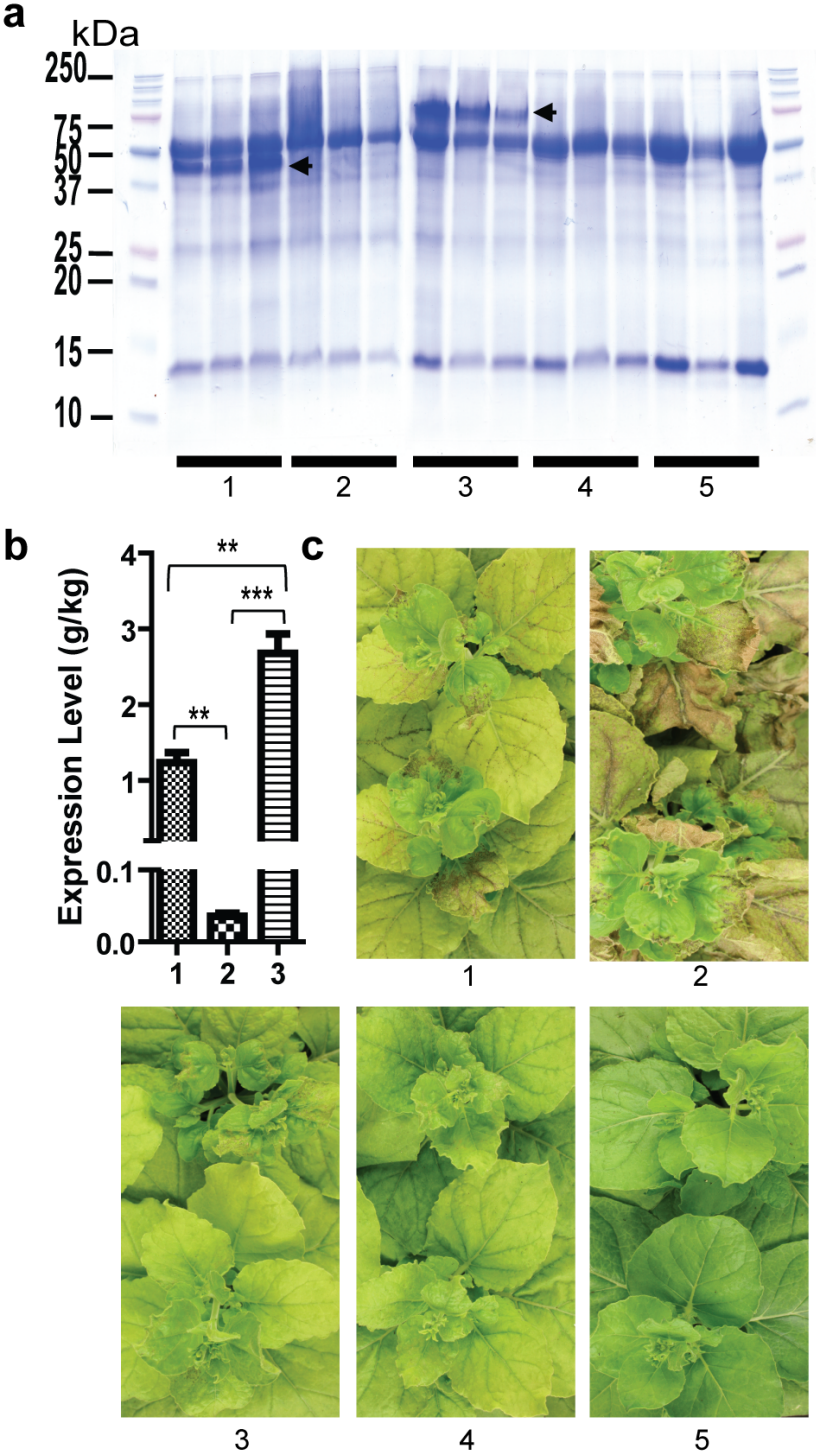
Comparison of gCTB, N4S-CTB, and N4S-CTB-KDEL at 5 dpi. (a) A Coomassie-stained non-denaturing SDS-PAGE resolving crude *N. benthamiana* leaf extracts. Numbers correspond to: 1. N4S-CTB-KDEL-expressing; 2. N4S-CTB-expressing; 3. gCTB–expressing; 4. empty vector-infiltrated; and 5. non-infiltrated plants, respectively, in biological triplicate (three independent plants). Arrowheads indicate N4S-CTB-KDEL and gCTB pentamers. (b) Quantification of CTB in leaf extracts at 5 dpi by GM1-ELISA. Numbers 1-3 correspond to N4S-CTB-KDEL, N4S-CTB and gCTB, respectively. Data are expressed as means ± SEM in biological triplicate. **P < 0.01, ***P < 0.001 (ANOVA with Bonferroni's multiple comparison test). (c) Photographs showing the phenotype of vector-inoculated plants at 5 dpi. Numbering is the same as in (a). Severe necrosis is evident with N4S-CTB and, to a lesser extent, N4S-CTB-KDEL, but not with gCTB.

In our previous study, we showed that N4S-CTB-KDEL accumulated up to 1 g per kg of fresh *N. benthamiana* leaf material using *Agrobacterium*-mediated delivery (agroinfiltration) of the magnICON tobamovirus replicon vector^26^. The “KDEL” ER retention signal was attached to the protein due to the *a priori* expectation of obtaining a high production yield. To revisit the role of ER retention in CTB biosynthesis and accumulation *in planta*, we performed site-directed mutagenesis to remove the codons coding for the C-terminal KDEL extension. The resulting variant, N4S-CTB, was expressed in *N. benthamiana* using the magnICON vector. Sodium dodecyl sulfate polyacrylamide gel electrophoresis (SDS-PAGE) analysis under non-denaturing conditions of crude leaf extracts, 5 days post vector inoculation (dpi), revealed no visible amount of N4S-CTB as compared to N4S-CTB-KDEL, which showed a clear band at around 60 kDa corresponding to the GM1-ganglioside receptor binding, pentameric form (Fig. 1a). A sensitive GM1-ganglioside-capture enzyme linked immunosorbent assay (GM1-ELISA) revealed that the receptor binding form of N4S-CTB was indeed expressed although the level was extremely low, i.e. approximately 50-fold lower than N4S-CTB-KDEL (Fig. 1b). Interestingly, the expression of N4S-CTB caused severe tissue damage in *N. benthamiana* plants at 5 dpi, while N4S-CTB-KDEL-expression induced only modest symptoms (Fig. 1c). These results suggested that ER retention played a critical role in the recombinant producibility of aglycosylated CTB and prevented tissue damage upon viral vector-based overexpression.

### Non-ER-retained but *N*-glycosylated CTB was accumulated at very high levels and did not cause tissue damage

Next, we explored the expression of the CTB construct, which contains an *N*-glycosylation site at Asn4 and lacks the C-terminal ER retention signal (gCTB). We hypothesized that *N*-glycosylation might facilitate folding and assembly of CTB via the help of lectin chaperones such as calnexin and calreticulin^11^,^12^,^29^. As expected, SDS-PAGE analysis demonstrated that gCTB was highly accumulated at 5 dpi in pentameric structures (Fig. 1a). *N*-Glycosylation of the protein was evident from the increased size compared with N4S-CTB-KDEL on an SDS-PAGE gel (Fig. 1a) and in immunoblot analyses using concanavalin A (ConA) and anti-horseradish peroxidase (HRP) antibodies, which recognize Xylose (Xyl)- and Fucose (Fuc)-containing plant glycans^30^ (Fig. S1c). GM1-ELISA data (Fig. 1b) showed that gCTB bound to the glycosphingolipid receptor and accumulated at exceptionally high levels in leaf tissue, i.e. up to 3.1 g per kg of fresh leaf material, which was significantly higher than the amounts obtained for N4S-CTB-KDEL (~1 g/kg). Notably, gCTB expression caused no tissue damage at 5 dpi, in sharp contrast to N4S-CTB. In fact, the morphological appearance of gCTB expressing plants was not different from that of empty vector-inoculated plants and was less pathologic than that of N4S-CTB-KDEL-expressing plants (Fig. 1c). Because virtually the only difference between gCTB and N4S-CTB is the presence or absence of *N*-glycosylation, this post-translational modification is likely responsible for the strikingly distinct outcomes in leaf morphology and recombinant production yield upon expression of these two CTB constructs.

To gain additional evidence supporting the above hypothesis, we created two N4S-CTB mutants that were engineered to be *N*-glycosylated at different Asn residues of the molecule: the Asn4→Ser, Lys23→Thr mutant (amino acid numbering based on mature CTB [Protein Data Bank ID: 1FGB]), which lost an *N*-glycosylation site at Asn4 but gained one at Asn21 (N4S-CTB-K23T); and another Asn4→Ser mutant, which gained an *N*-glycosylation site at the C-terminal Asn106 by adding a C-terminal extension of a Val-Thr-Lys-Ala-Leu-Leu sequence following the Asn residue (thus Asn106-Val107-Thr108 constitutes an *N*-glycosylation sequon; N4S-CTB-VTKALL). These proteins were expressed in *N. benthamiana* leaves using the magnICON vector. We found that both N4S-CTB variants accumulated at relatively high levels at 5 dpi, with 1.0 g/kg for the former and 1.19 g/kg for the latter variants (Fig. S1a), and most importantly, induced hardly any leaf tissue damage as with gCTB (Fig. S1b). ConA- and immuno-blot analysis demonstrated that these two N4S-CTB variants were indeed glycosylated (Fig. S1c).

Taken together, the above results clearly indicate that *N*-glycosylation alleviates tissue damage induced by the overexpression of CTB and facilitates accumulation of the properly assembled protein in plants.

### *N*-Glycosylation reduced ER stress and the hypersensitive response (HR)

**Figure 2:**
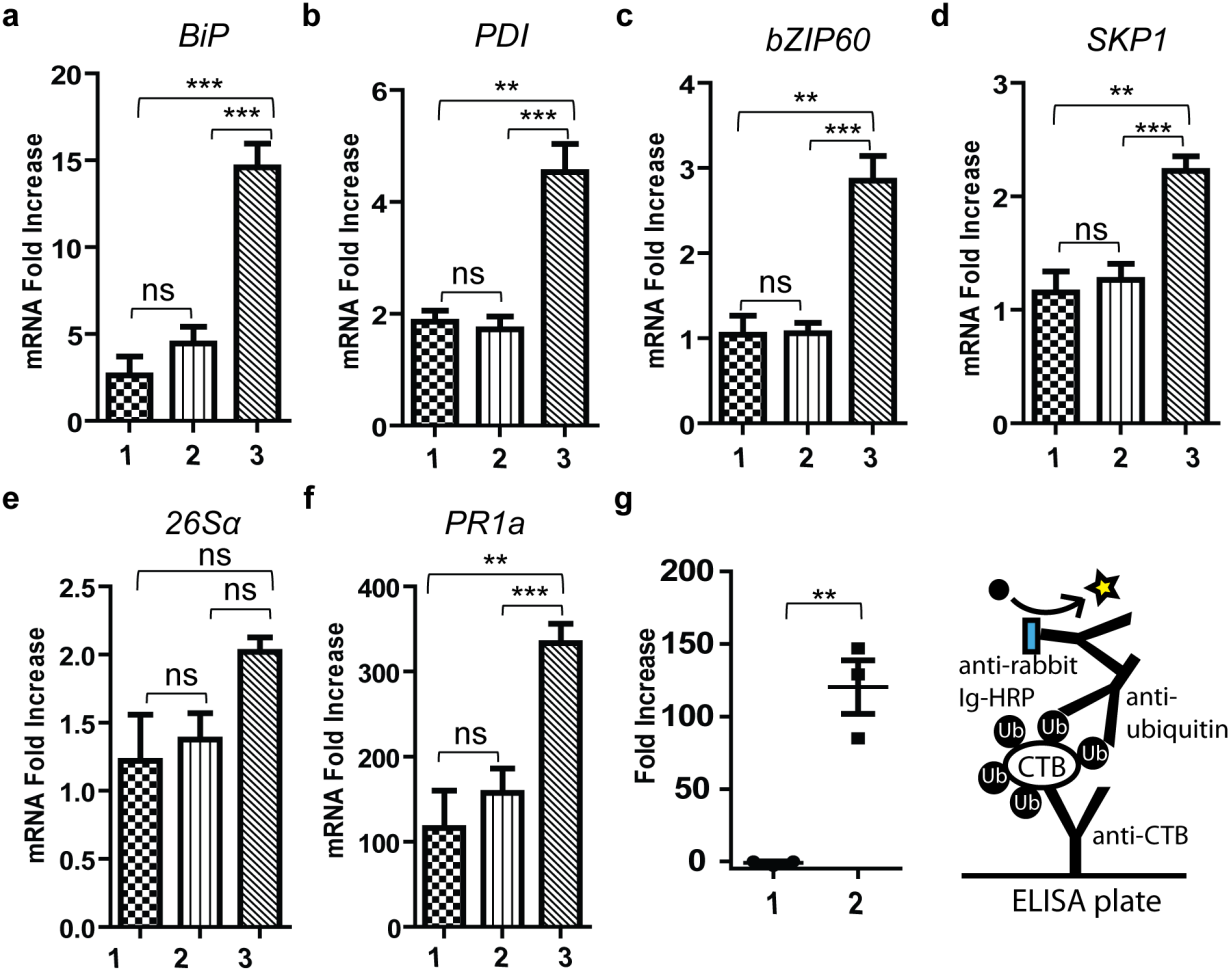
Relationship between stress response and *N*-glycosylation in CTB overexpression. RT-qPCR analysis of stress genes: (a) *BiP* (b) *PDI* (c) *bZIP60* (d) *SKP1* (e) *26Sα* (f) *PR1a*. RNA was extracted from leaf tissues at 2 dpi. Starting amounts of cDNA for each gene were normalized to that of *18S* ribosomal RNA (reference gene). Data were plotted as fold increase to the average normalized value for non-infiltrated plants and are expressed as means ± SEM of biological replicates (n = 9). **P < 0.01, ***P < 0.001, ns: not significant, one-way ANOVA with Bonferroni's multiple comparison test. Numbers 1-3 correspond to empty vector-infiltrated, gCTB-expressing and N4S-CTB-expressing plants, respectively. (g) Left: Detection of ubiquitinated CTB in leaf extracts at 3 dpi by ELISA. Data were plotted as fold increase to the average value for empty vector infiltrated plants and are expressed as means ± SEM (n = 3). **P < 0.01; unpaired two-tailed t-test. Numbers 1 and 2 correspond to gCTB- and N4S-CTB-expressing plants, respectively. Right: Schematic depicting ubiquitin ELISA.

The above observation prompted us to analyze the mechanism by which *N*-glycosylation relieved leaf damage and led to high gCTB accumulation. We found that tissue damage induced by N4S-CTB expression began between 2 and 3 dpi, while no sign of stress was observed for gCTB up to 5 dpi; gCTB accumulation reached maximum at 5 dpi and declined thereafter with no phenotypic change in the tissue (data not shown). Accumulation levels of GM1-ganglioside-binding N4S-CTB and gCTB in leaf tissue at 3 dpi were 0.067 and 0.38 g/kg, respectively. Hence, poor accumulation of N4S-CTB had already taken place at an early stage of protein expression, which appeared to be accompanied by tissue damage. These findings suggested that inefficient folding and/or assembly of the non-glycosylated protein might have caused plant stress. Thus, we measured the expression of representative stress markers, i.e., *PDI*, *BiP*, *bZIP60*, *SKP1*, *26S* proteasome and pathogenesis-related protein 1a (*PR1a*), by reverse-transcription quantitative real time (RT-q)PCR in leaf tissues-expressing N4S-CTB and gCTB at 2 dpi. The results demonstrated that N4S-CTB-expressing plants displayed a statistically significant increase in transcript levels of *PDI*, *BiP*, *bZIP60*, *SKP1* and *PR1a* compared with control plants infected with empty vector (*P* < 0.01 or < 0.001 as compared to the empty vector control; 1-way ANOVA followed by Bonferroni's multiple comparison test), whereas gCTB-expressing plants showed no increase in expression of these genes (Fig. 2). Although not statistically significant, the *26*S*α* gene also showed an increased expression trend with N4S-CTB but not with gCTB. Given that *PDI*, *BiP* and *bZIP60* are up-regulated during UPR in plants^13^,^14^, these results suggest that N4S-CTB induced strong UPR and ER stress, while *N*-glycosylation of the protein completely prevented this response. Consistent with the RT-qPCR data, ubiquitin ELISA showed that N4S-CTB polypeptides were significantly more ubiquitinated than gCTB at 3 dpi (Fig. 2g); ubiquitinated proteins were not detected at 2 dpi for either construct (data not shown). The increase of *SKP1* and *26*S*α* gene expression along with the increase of ubiquitination point to the degradation of misfolded and/or unassembled N4S-CTB polypeptides by the ERAD pathway^13^.

Unlike other genes examined, the expression of *PR1a*, a marker for HR induced by pathogen infection^31^,^32^, was increased by over 100-fold in all three groups (i.e., empty vector-infiltrated, and gCTB- and N4S-CTB-expressing plants) compared to non-infiltrated control tissue. This might be explained by the tobamoviral vector replication and/or *Agrobacterium* used for vector delivery. Interestingly, N4S-CTB-expressing leaf tissue showed significantly more *PR1a* gene expression than the other two tissues (*P* < 0.01), suggesting the potential contribution of HR along with ER stress to the tissue damage induced by the non-glycosylated protein expression.

### gCTB maintains nanomolar receptor binding affinity and physicochemical stability that are indicative of *in vivo* immunological activity

The finding that GM1-ganglioside receptor-bindable gCTB was produced at remarkably high levels in *N. benthamiana* stimulated our interest in investigating whether the protein may be developed as a novel vaccine antigen. Thus, we purified gCTB to characterize its molecular properties relevant for vaccine use. We employed the extraction and purification protocols previously developed for N4S-CTB-KDEL^26^, whereby gCTB was purified with a 2-step chromatography process: immobilized metal affinity chromatography, followed by ceramic hydroxyapatite. An overloaded non-denaturing SDS-PAGE demonstrated that pentameric gCTB was purified to >95% homogeneity (data not shown).

**Figure 3:**
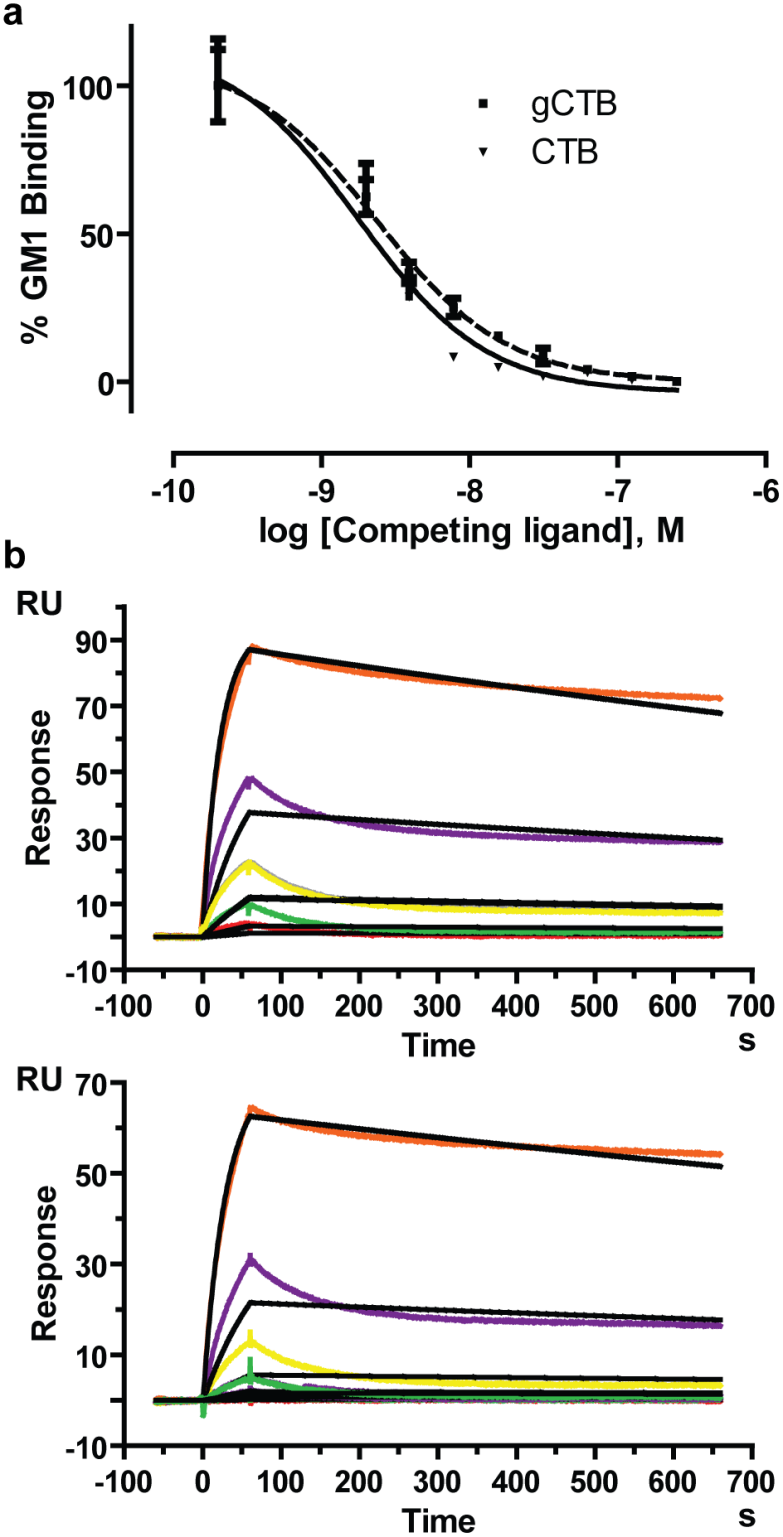
GM1-ganglioside-binding activity of gCTB. (a) Binding affinity determination based on competitive GM1-ELISA. The assay was performed in triplicate. The 50% inhibitory concentrations (IC_50_) of native CTB and gCTB were determined to be 1.8 and 2.4 nM, respectively. (b) Surface Plasmon Resonance. Each CTB protein was immobilized on a sensor chip, and GM1-ganglioside was used as the analyte. Representative sensorgrams obtained with gCTB (Top) and native CTB (Bottom) are shown. The capture level of gCTB was 210 RU and of native CTB was 175 RU. The colored curves represent various concentrations of GM1-ganglioside (10, 3.33, 1.11, 0.37, and 0.123 μg/ml from top to bottom), and the black lines are the 1:1 binding kinetics fit. The *K*_D_ values for gCTB and CTB were determined to be 60.1 ± 1.7 and 51.4 ± 5.7 nM, respectively (means ± SD of triplicate).

First, we investigated the GM1-ganglioside binding affinity of gCTB because it is the most essential attribute for vaccine efficacy. A competitive GM1-ELISA (Fig. 3a) revealed that gCTB showed a similar affinity to GM1-ganglioside as a *Vibrio cholerae*-derived commercial CTB (Sigma-Aldrich, St. Louis, MO); CTB and gCTB showed 50% inhibitory concentrations (IC_50_) of 1.8 and 2.4 nM, respectively. Surface plasmon resonance was performed to determine the equilibrium dissociation constants (*K*_D_) of these molecules (Fig. 3b). Employing the Biacore X100 1:1 binding kinetic analysis, we found that CTB and gCTB had *K*_D_ values of 51.4 ± 5.7 and 60.1 ± 1.7 nM, respectively, not statistically significant as determined by unpaired two-tailed t-test (*P* > 0.05).

**Figure 4:**
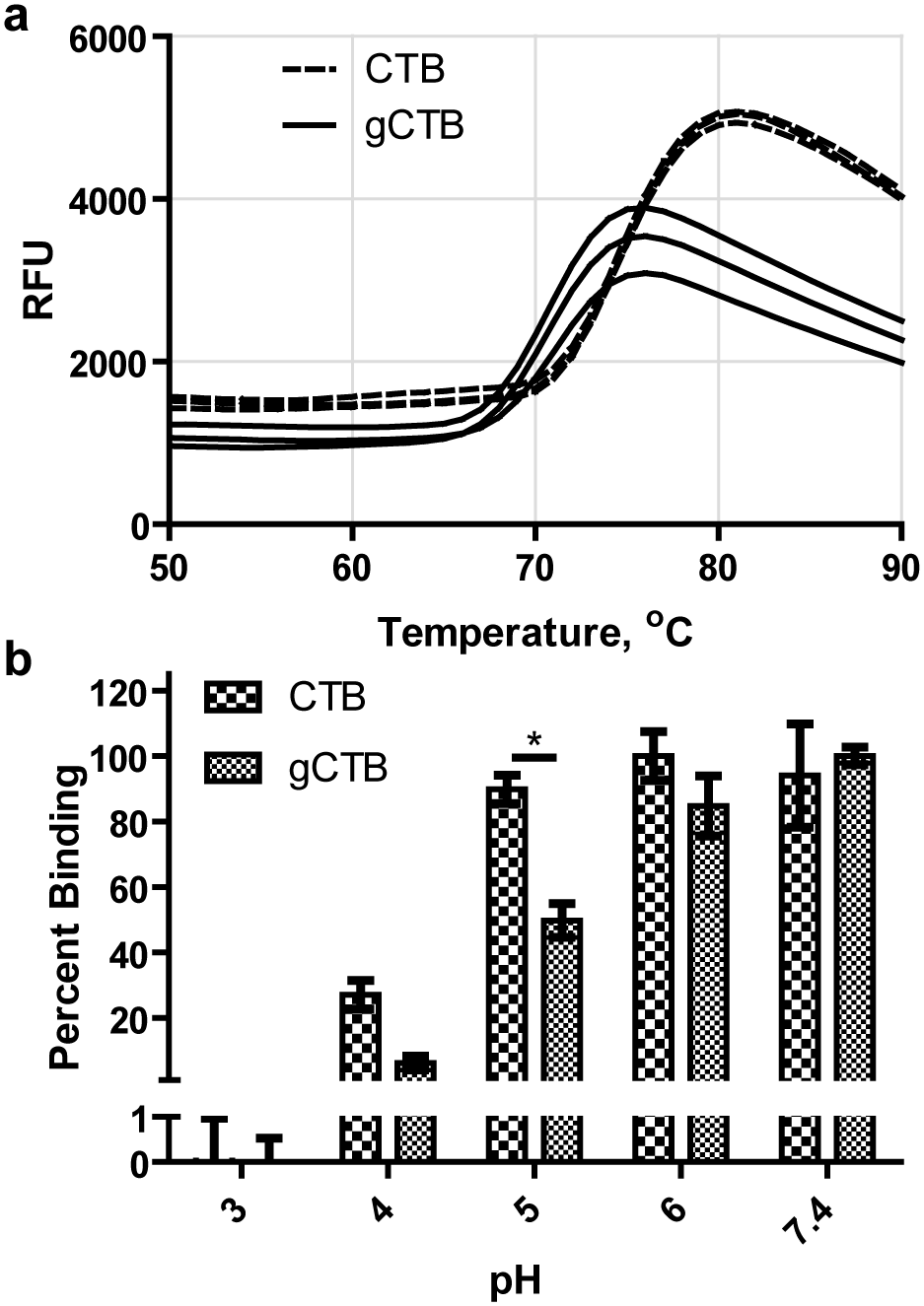
Thermal and acid stability of gCTB. (a) Melting temperature (*T*_m_) determination using DSF. CTB (dashed line, n = 3) and gCTB (solid line, n = 3) were analyzed. *T*_m_ values of CTB and gCTB were 74.2 and 70.9°C, respectively, as determined by the vertex of the first derivative of relative fluorescence unit (RFU) values. (b) pH stability analysis. CTB and gCTB were incubated in various pH buffers^26^, and GM1-bound CTB proteins were quantified by GM1-ELISA. The results were normalized to the average value of the corresponding CTB protein at pH 7.4 and expressed as % binding. Data represent means ± SEM (n = 3); * *P* < 0.05, unpaired two-tailed t-test.

Next, we analyzed melting temperatures (*T*_m_) to assess potential structural instability associated with *N*-glycosylation of CTB. Using differential scanning fluorimetry (DSF), CTB and gCTB showed *T*_m_ values of 73.93 ± 0.23 and 70.47 ± 0.31°C, respectively (Fig. 4a). Thus, gCTB had slightly lower thermostability than the native counterpart. This trend was observed across various pH conditions studied, with more pronounced impacts observed at lower pH (Table 1). To confirm these findings, we performed a GM1-ELISA using the proteins exposed to varying pH conditions (Fig. 4b). The results again showed that gCTB is less acid stable than CTB; at pH 5 the glycosylated protein lost approximately half of GM1-ganglioside binding activity while the non-glycosylated native protein retained over 90% activity, as compared to their respective receptor-binding activities at pH 7.4.

Taken together, we concluded that the Asn4 glycosylation of CTB does not impinge on GM1-ganglioside binding affinity but marginally affected physicochemical stability as revealed at fine analytical levels by the biophysical and biochemical methods used here. Nevertheless, the results suggest that gCTB may maintain critical molecular properties for immunological activity under physiological conditions, given the nanomolar GM1-ganglioside binding affinity and >70°C *T*_m_ at neutral pH.

### gCTB displays a heterologous population of complex N-glycans including plant specific glycoforms

For the development of biopharmaceuticals based on recombinant glycoproteins, it is imperative to analyze their glycan compositions as part of the product profile. First, we characterized the *N*-glycosylation of gCTB at the whole molecule level. Heat-denaturing SDS-PAGE (Fig. S1c) revealed two forms of monomeric gCTB at 14.5 kDa and 12.5 kDa. Based on a densitometric analysis, it was estimated that the ratio of the high- and low-molecular-weight monomers were 77.2:22.8. A ConA blot and an anti-HRP immunoblot showed that only the upper band of gCTB was modified with *N*-glycans including plant-specific glycoforms (Fig. S1c), which in turn explained that the ~2kDa difference between the two monomeric forms was due to the presence or absence of an *N*-glycan. Consequently, 22.8% of the nascent gCTB polypeptide was not *N*-Glycosylated. Such incomplete *N*-glycosylation site occupancy was previously observed for an ER-retained gCTB variant expressed in transgenic *N. benthamiana*^26^ and transgenic rice seeds^20^. Also, this is not uncommon for glycoproteins produced in various recombinant systems^33^,^34^,^35^.

**Figure 5:**
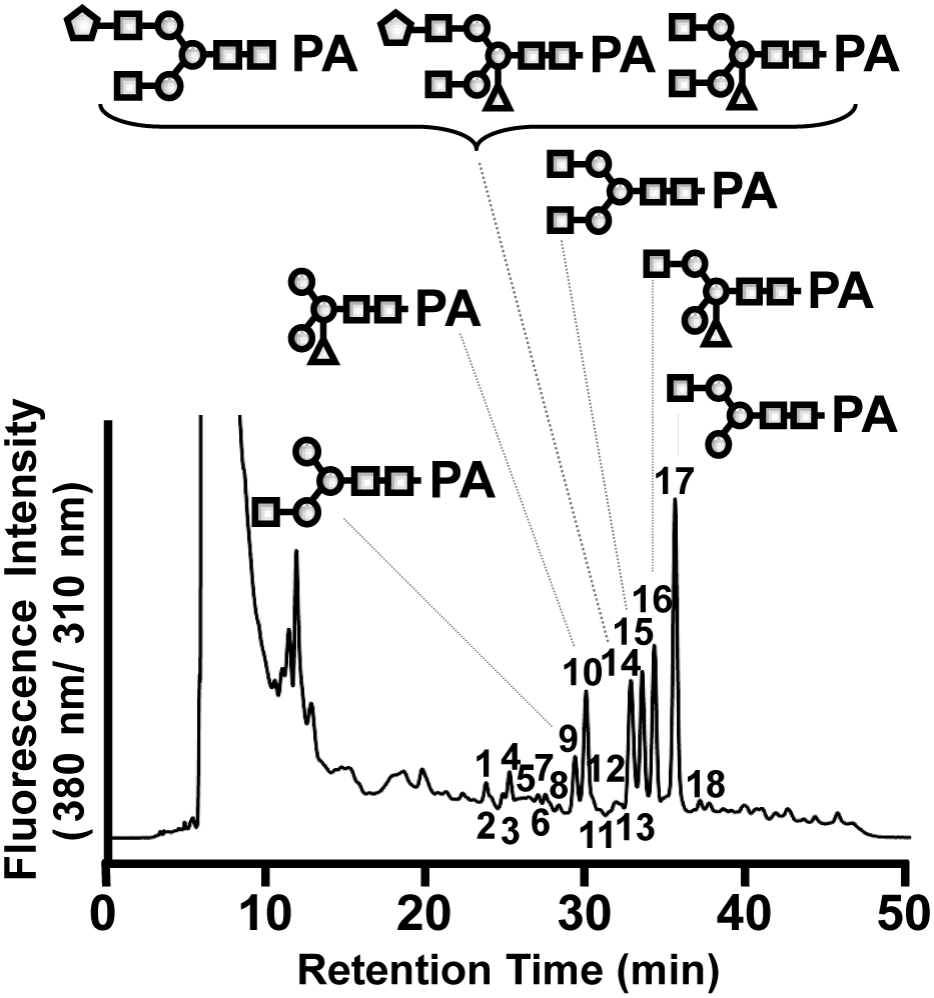
Glycan profile of gCTB. The chromatogram shows RP-HPLC separation of PA-labeled glycans isolated from gCTB. Glycan structures with >2% relative abundance are depicted at corresponding PA-glycan fractions. Symbols: circle (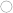), mannose; square (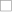), N-acetylglucosamine; pentagon (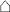), β1,3-galactose; diamond (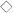), fucose and triangle (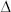), xylose.

To further dissect the *N*-glycan profile of gCTB, we analyzed the glycan composition and structure using a combination of comparative high-performance liquid chromatography (HPLC) and mass spectrometry (MS). Eighteen distinctive fractions of 2-aminopyridine (PA) derivatives were identified in reversed-phase (RP)-HPLC (Fig. 5), each of which was subsequently subjected to size-fractionation (SF-)HPLC for further separation (Fig. S2, S3; fractions 1–18). Fig. 5 shows representative glycan structures depicted at corresponding PA-glycan fractions with greater than 2% relative amount of total glycans found. The *N*-glycans attached to gCTB were composed of various complex-type glycoforms, including those containing plant-specific β(1,2)-linked Xyl, one or two terminal N-acetylglucosamine (GlcNAc) and/or a terminal β(1,3)-linked galactose (Table 2). This glycan profile indicated that gCTB was secreted through the endomembrane system, as expected^36^,^37^. Specifically, the results suggest that gCTB mostly traversed through the Golgi apparatus given that more than half of the glycans were complex *N*-glycans. Interestingly, unlike ER-retained gCTB expressed in transgenic *N. benthamiana*^26^, gCTB contained few α(1,3)-linked Fuc, another plant-specific glycan structure. In fact, a significant fraction (>50%) of these glycans were shown to be devoid of plant-specific glycan modifications.

### gCTB retains oral immunogenicity to induce mucosal and systemic antibody responses

**Figure 6:**
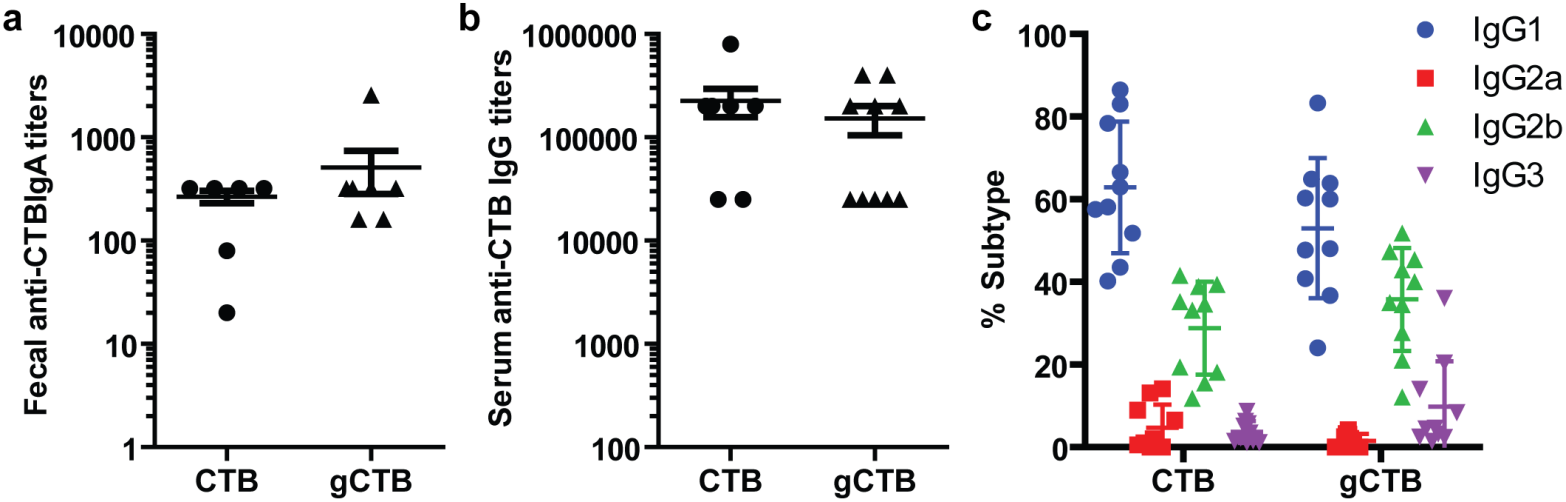
Oral immunogenicity of gCTB. Anti-CTB antibody titers. C57bl/6 mice were orally immunized twice with PBS, gCTB, or CTB (3 μg). (a) Fecal anti-CTB SIgA, (b) Serum anti-CTB IgG and (c) Serum IgG subtype. Endpoint titers were analyzed 2 weeks after the second immunization. Horizontal bars show mean titers, whereas symbols represent titers of individual mice (for serum IgG) or pooled analysis (for fecal IgA; fecal samples of each group were pooled for analysis). There was no significant difference between total endpoint titers induced by CTB and gCTB (*P* > 0.05;2-tailed unpaired t-test).

To examine the vaccine potential of gCTB, we assessed its oral immunogenicity in mice. *V. cholerae*-derived CTB was used as a control in these experiments, where 3 µg of gCTB or CTB were orally administered to mice followed by an oral boost using the same dosage 2 weeks after the initial immunization, following a standard regimen used for the Dukoral® cholera vaccine^38^. This dosage was chosen to effectively detect any potential impact on immunogenicity caused by CTB glycosylation, as our preliminary study has shown that antibody titers reach maximum with ≥10 µg of CTB (data not shown). ELISA was employed to determine cholera holotoxin-binding antibody titers. As shown in Fig. 6a, both the glycosylated and native proteins elicited comparable anti-holotoxin IgA levels in fecal extracts, with average titers of 512 ± 229 and 266 ± 36, respectively. Likewise, gCTB and CTB induced comparable (not statistically different; *t*-test) serum anti-holotoxin IgG levels, with average titers of 152,500 ± 48,369 and 225,000 ± 67,905, respectively (Fig. 6b). Further dissection of the systemic antibody response revealed that gCTB and CTB induced overall similar humoral immunity with comparable IgG subclass distribution; the majority (≥50%) were IgG1, followed by IgG2b (30–40%) (Fig. 6c), which is in accordance with a Th2-dominated response previously demonstrated for CTB^39^,^40^. These data demonstrate that gCTB retains oral immunogenicity comparable to the original protein and CTB glycosylation does not seem to affect the type of immunity induced by the protein.

## Discussion

Plant-based recombinant protein expression systems provide a viable option for the production of pharmaceutical proteins. In particular, transient overexpression systems based on plant viral and non-viral vectors offer rapid, robust and scalable bioproduction. Although both glycosylated and non-glycosylated forms of CTB have previously been produced in several different plant platforms^19^,^20^,^21^,^22^,^23^,^24^,^25^,^41^,^42^, our transient CTB mass production system may obviate the need for a vaccine stockpile while enabling the implementation of reactive vaccination against cholera outbreaks. This has significant implications for cholera prevention strategies because the efficacy of currently available cholera vaccines lasts only a few years^38^,^43^,^44^.

Previously, we reported that an ER-retained non-glycosylated CTB variant (N4S-CTB-KDEL), a vaccine antigen against cholera, accumulated to high levels in *N. benthamiana*^26^. It has been empirically shown that ER retention generally provides high protein accumulation in plants. Indeed, herein we found that the overexpression of N4S-CTB by agroinfiltration of the magnICON tobamoviral replicon vector brought about a marginal yield (Fig. 1b) and the plants suffered from massive tissue damage (Fig. 1c; number 2). However, when *N*-glycosylated, the non-ER-retained protein accumulated at high levels and caused no tissue damage (Fig. 1, S1). Up to 3.1 g of gCTB was obtained per kg of fresh leaf material (Fig. 1b), which was significantly higher than the amounts described for N4S-CTB-KDEL^26^ and arguably among the highest achieved for plant-made biopharmaceuticals to date. Given that the introduction of *N*-glycosylation to Asn21 or Asn106 of N4S-CTB resulted in similar phenotypes observed with gCTB, i.e. increased protein production (Fig. S1a) and little tissue damage at 5 dpi (Fig. S1b), it is concluded that *N*-glycosylation in general, albeit with varying degrees of efficacy depending on its locus, can improve the bioproducibility of CTB in *N. benthamiana*. These findings provide important implications for transgene design to improve recombinant production yields while alleviating plant stress.

Recent reports have shown that recombinant proteins expressed in transgenic rice^8^,^14^,^15^ and an antibody expressed in Arabidopsis seeds^45^ induced UPR. Furthermore, changing BiP amounts to an optimal level in transgenic rice leads to increased protein production^46^. To the best of our knowledge, this is the first report investigating ER stress during foreign protein production in *N. benthamiana*. Given that virus-based transient production of recombinant proteins in *N. benthamiana* is most progressive, information leading to the mechanism of failed UPR, ERAD and PCD can lead to healthier plant conditions and increase protein accumulation. In RT-qPCR analysis (Fig. 2), N4S-CTB, but not gCTB, caused a significant up-regulation of the UPR genes *BiP*, *bZIP60* and *PDI*. Also, the ERAD genes *SKP1* and *26Sα* were up-regulated by N4S-CTB. These findings suggest that N4S-CTB expression caused ER stress, inducing both the UPR and ERAD pathways. Thus, misfolded and unassembled N4S-CTB polypeptides that could not be rescued by molecular chaperones were eventually ubiquitinated, as shown in Fig. 2g, and degraded by ERAD. Due to the intense protein synthesis driven by the viral vector, the payload may have exceeded the capacity of ERQC and ERAD systems, therefore imposed severe stress on the host plant and finally resulted in the observed tissue damage. Of interest is the fact that N4S-CTB expression also led to a significant increase of *PR1a* expression. This may imply that the tissue damage is not solely attributed to ER stress; it might represent the complex interplay of ER stress and HR induced by the overflow of misfolded proteins and *Agrobacterium*-mediated viral vector delivery. Meanwhile, the introduction of *N*-glycosylation to N4S-CTB has dramatically increased the yield of properly assembled pentameric proteins (Fig. 1b and S1a) likely via the assistance of lectin chaperones, which in turn minimized ubiquitination (Fig. 2g), stress marker gene expression (Fig. 2a-f) and tissue damage (Fig. 1c and S1b). The above scenario can also explain why functional N4S-CTB-KDEL was produced at relatively high levels with moderate tissue damage when the KDEL signal peptide was attached to the C-terminus (Fig. 1), as the chance of proper folding/assembly would have been improved by a prolonged residence time in the ER^10^,^12^ without the help of the *N*-glycan dependent calnexin/calreticulin protein folding pathway^13^. Taken together, our data indicate that the structural formation of CTB is a relatively inefficient process in plant cells, and hence *N*-glycosylation or ER retention is indispensable for the efficient bioproduction of the vaccine antigen in the present system. To our knowledge, this is the first report shedding light on UPR, ERAD and tissue damage caused by transient overproduction of a recombinant protein by agroinfiltration of viral vectors in *N. benthamiana*.

Plant complex-type *N*-glycans are structurally different from their mammalian counterparts. Plant glycans often contain β(1,2)-Xyl not found in mammals, and have α(1,3)-Fuc attached to the core N-acetylglucosamine (GlcNAc) instead of α(1,6)-Fuc found in mammals. In addition, β(1,3)-galactose and α(1,4)-Fuc are found at the terminal GlcNAc in plants in lieu of β(1,4)-galactose in mammals^37^. The Asn4-attached glycan on gCTB expressed in *N. benthamiana* showed a distinct profile comprised of approximately 40% β(1,2)-Xyl but only 0.8% Fuc (Fig. 5; Table 2). Although the underlying mechanism for the relatively low fucosylation is unclear, these findings suggested that gCTB was efficiently excreted through the endomembrane system with little to no protein retained in the ER, since there were few (<5%) high-mannose-type glycans. The gCTB glycan profile is different from, and perhaps more uniform than, that of the ER retained gCTB expressed in transgenic *N. benthamiana*, which was broadly distributed throughout the endomembrane system and contained complex as well as high-mannose-type glycans^26^. In addition, CTB containing KDEL expressed in transgenic rice was not sufficiently retained in the ER and contained approximately 50% plant-specific glycan structures^20^.

Notwithstanding the slightly reduced thermo- and acid-stability of gCTB as demonstrated in our biochemical and biophysical analyses (Fig. 4), the added *N*-glycans did not significantly affect pentamer formation (Fig. 1a) or the GM1-ganglioside binding affinity (Fig. 3) of the protein, the critical characteristics attributed to CTB's immunostimulatory activity. It should be mentioned that the SPR sensorgrams for each CTB showed higher experimental Rmax values than the calculated Rmax. The high experimental Rmax is hypothesized to be attributed to micelle formation of GM1-ganglioside given that the higher concentrations are acting as a mixed species (soluble+micelle GM1). To address this issue, analysis was performed with GM1 pentasaccharide^47^, which does not form micelles, unlike GM1-ganglioside. With GM1 pentasaccharide the experimental Rmax values were not greater than the calculated Rmax, equilibrium was reached, and there was good agreement of fitted curves to experimental curves (data not shown).

gCTB was highly immunogenic in mice after oral administration (Fig. 6), inducing comparable anti-cholera holotoxin IgA, IgG and IgG subtype titers as native CTB. Thus, we propose that from the vaccine efficacy standpoint gCTB can serve as an alternative to bacterial and other plant-made CTB^19^,^20^,^21^,^22^,^23^,^24^,^25^,^41^,^42^ towards mass vaccination against cholera. Plant-specific glyco-epitopes containing β(1,2)-Xyl and α(1,3)-Fuc residues are known to be immunogenic in most mammals^48^. Nevertheless, potential immunotoxicity in humans is difficult to examine in animal models and can only be ruled out in clinical investigations^49^,^50^. In case that gCTB induces such an adverse effect due to plant-specific glycans, protein production in a transgenic *N. benthamiana* line devoid of xylosyl- and fucosyltransferases (e.g., see Strasser et al.^30^) would provide a solution. Significant progress has been and is currently being made towards the so called humanization of protein *N*-glycosylation in plant cells^37^,^51^. Apart from potential use in cholera vaccines, the *N*-glycan of gCTB could be deliberately exploited to expand the protein's utility; for example, plants could be forced to produce high-mannose-type glycans that are common between plant and mammalian cells, as illustrated by gCTB produced in *N. benthamiana* hydroponically treated with kifunensine (Kif-gCTB) (see Fig. S4). Kifunensine is an alkaloid that inhibits class I α-mannosidases including plant mannosidase I, causing a complete shift in the structure of the *N*-glycans from complex forms to the Man_9_GlcNAc_2_ high-mannose-type oligosaccharide, when placed in medium at concentrations of 1 µg/ml or higher^62^. It is noteworthy that using flow cytometry we found that gCTB and Kif-gCTB bound to the C-type lectin receptor Dendritic Cell-Specific Intercellular adhesion molecule-3-Grabbing Non-integrin (DC-SIGN) on the surface of Raji cells, with the latter displaying a significantly higher affinity (Fig. S5). DC-SIGN recognizes a large array of viral, bacterial, fungal and parasitic pathogens in an oligosaccharide-dependent manner and may be utilized in vaccine development^52^,^53^. Hence, although there seemed to be no difference in the humoral immune responses induced by gCTB and native CTB upon oral vaccination (Fig. 6a and b), the possibility remains that gCTB exerts an additional immunomodulatory effect via the lectin receptor of antigen presenting cells when these cells are effectively targeted through an appropriate vaccination route. Future studies are warranted to determine the significance of DC-SIGN binding by gCTB, which might open new avenues for the use of the highly bioproducible immunostimulatory protein in research and vaccine development.

## Methods

### Vector construction and expression of CTB in *N. benthamiana*

The magnICON tobamovirus replicon system^54^,^55^ was used. The synthetic *CTB* sequence (GenBank accession no. AY475128) with an 18-nucleotide extension (TCCGAGAAGGATGAACTC) at the 3′ end that encodes the C-terminal SEKDEL sequence^27^,^56^ was sub-cloned into the magnICON vector pICH11599 to generate pNM47. PCR was used to remove the *V. cholerae* secretory signal from the original *CTB* gene, using pNM47 as a template. The PCR product was sub-cloned into pICH11599 to generate pNM134. Site directed mutagenesis was performed using the Quikchange II Site-Directed Mutagenesis Kit (Agilent Technologies, Santa Clara, CA) according to the manufacturer's instructions, with pNM134 as template and primers that mutated the nucleotide A at position 74 (GenBank accession no. AY475128) to G, creating pNM156 for the Asn4→Ser CTB variant, N4S-CTB-KDEL^26^. Site directed mutagenesis was also used to remove the C-terminal SEKDEL. Using pNM134 and pNM156 as templates and primers that removed the nucleotides coding for SEKDEL, pNM160 (gCTB) and pNM161 (N4S-CTB), respectively, were created. DNA sequences for N4S-CTB-K23T and N4S-CTB-VTKALL were *de novo* synthesized based on the *CTB* sequence (Integrated DNA Technology, San Diego, CA). The two variants were sub-cloned into pICH11599 to generate pNM307 and pNM326, respectively.

CTB proteins were expressed with the rice α-amylase signal peptide based on our previous findings^26^. For this purpose, the 5′ provector pICH20155 was used with the 3′ provector pICH14011 and pNM156, pNM160, pNM161, pNM307, or pNM326. As an empty vector control, pICH20155, pICH14011, and pICH11599 were used. The vectors were delivered into *N. benthamiana* leaves using the vacuum agroinfiltration method^57^. After 5 days, clarified leaf extracts were generated as previously described^26^ and analyzed for CTB expression by SDS-PAGE and GM1-ELISA as described previously^58^.

### Purification of CTB

N4S-CTB-KDEL, gCTB, N4S-CTB-K23T, and N4S-CTB-VTKALL were purified from leaf extracts as previously described^26^. The concentrations of purified proteins were determined spectrophotometrically at 280 nm, using their theoretical extinction coefficient.

### Ubiquitin ELISA

Leaf material was extracted in 1x SDS sample buffer with a 2:1 buffer to tissue ratio using a Precellys homogenizer, then centrifuged twice (13,000 × g, 10 minutes) generating crude leaf extracts. A polystyrene plate was coated with goat anti-CTB antibodies (List Biological, Campbell, CA), blocked with PBSTM and crude leaf extracts were added. Ubiquitinated CTB proteins were probed with rabbit anti-ubiquitin antibodies (AgriSera, Vännäs, SWEDEN) followed by HRP-conjugated goat anti-rabbit antibodies (Santa Cruz Biotechnology, Dallas, TX). Finally, ubiquitinated CTB-antibody complexes were detected using TMB substrate (Surmodics, Eden Prairie, MN) and reading absorbance at 450 nm.

### Analysis of N-glycans attached to gCTB expressed in *N. benthamiana*

Glycan profiling was performed as previously described^26^,^27^.

### Biochemical and biophysical analysis of gCTB

Competitive GM1-ELISA was performed as described previously^27^ except that HRP-CTB (Molecular Probes, Grand Island, NY), at the concentration of 2 µg/ml, was used as a competitor. The *K*_D_ of GM1 ganglioside to CTB was measured using a Biacore X100 2.0 instrument, the *T*_m_ and acid stability of CTB were determined by differential scanning fluorimetry (DSF), and acid stability was also determined by GM1-ELISA as previously described^26^. Native CTB (Sigma-Aldrich) was used as a reference control.

### RNA extraction, reverse transcription, RT-qPCR

RNA was extracted from 100 mg of fresh plant leaf material (Non-infiltrated plants [n = 3]; Empty vector-infiltrated, gCTB-expressing and N4S-CTB-expressing plants [n = 9]), 48 h post-infection, by disrupting the tissue using liquid nitrogen and a mortar and pestle followed by QIAShredder and RNeasy Mini Kits (Qiagen, Venlo, The Netherlands). The provided protocol for Plants and Fungi was used, utilizing the optional on-column DNase digestion, to eliminate genomic DNA. RNA samples were eluted in 50 µL RNase-free water and nanodropped (Nanodrop Spectrophotometer ND-100, Thermo Scientific, Wilmington, DE) to determine concentration. All RNA samples were pure as determined by having OD_260/280_ and OD_260/230_ ratios greater than 2. RNA was immediately stored at -80°C and used within 1 month. For reverse transcription, first strand cDNA was synthesized using the RNA PCR Kit (AMV) Version 3.0 kit (Takara, Otsu, Shiga, Japan), step A only. The reaction (10 μl) was performed at 42°C for 1 h using 500 ng RNA, AMV reverse transcriptase and the provided random 9-mers. cDNA was diluted 1:10, stored, in aliquots, at -20°C and used within 2 months. RT-qPCR was performed on an iQ5 Multicolor Real-Time PCR Detection System (Bio-Rad, Hercules, CA) using iQ SYBR Green Supermix (Bio-Rad). The provided Bio-Rad iQ protocol was followed for a final reaction volume of 20 µL, including master mix, primers (100 nM) and 5 µL of cDNA. Supplementary Table 1 summarizes the primers and amplicon characteristics. The starting amounts of cDNA for each gene were normalized to the reference gene, *18S* ribosomal RNA^59^,^60^ and normalized values were plotted as fold increase to non-infiltrated plants. The expression stability of *18S* was evaluated using two established methods, NormFinder^61^ and BestKeeper^62^. NormFinder demonstrated stability values of 0.009, 0.019 and 0.034 for *18S*, *F-Box* and *PP2A*, respectively. BestKeeper analysis is shown in Table S2. Both methods found *18S* to be the highest ranked reference gene.

### Animal housing, immunization, sample collection, quantification of mucosal and systemic Igs

Eight week-old female C57BL/6J mice were purchased from The Jackson Laboratory in Bar Harbor (Maine, USA). Four animals were housed in each cage in a temperature- and humidity-controlled room with alternating light/dark cycles of 12 h, with access to Laboratory Autoclavable Rodent Diet 5010 (LabDiet, St. Louis, MO) and water *ad libitum*. All experimental procedures were approved by the University of Louisville's Institutional Animal Care and Use Committee. Mice were acclimated for approximately one week prior to the initiation of the studies. Mouse immunization, sample collection and quantification of mucosal and systemic Igs were performed as previously described^26^.

### Statistical analyses

Statistical significance was analyzed by one-way ANOVA with Bonferroni's multiple comparison test or student's *t*-test unless otherwise stated, using the GraphPad Prism 5.0 software. Differences were considered statistically significant if *P* < 0.05.

## Author Contributions

N.M. and K.T.H. conceived of and designed the study. K.T.H., J.C.K., J.M.J., B.N., L.J.M., A.S.H. and H.K. performed experiments. K.T.H., J.C.K., A.S.H., H.K., K.F. and N.M. analyzed data. K.T.H., J.C.K. and N.M. wrote the manuscript. All authors reviewed the manuscript.

## Supplementary Material

Supplementary Information Supplementary figures and legends

## Figures and Tables

**Table 1:** pH stability of CTB and gCTB determined by DSF^a^

	*T*_m _(°C)^b^	
pH	gCTB	CTB
3.0	23.0 ± 0	40.4 ± 0
4.0	58.8 ± 2.5	66.7 ± 1.4
5.0	67.0 ± 0.7	77.3 ± 0.3
6.0	72.5 ± 1.0	80.6 ± 1.4
7.4	70.5 ± 0.8	73.9 ± 1.2

^a^Proteins were diluted in appropriate pH buffers (see Experimental Procedures for details).

^b^*T*_m_ were determined by the vertex of the first derivative of relative fluorescence unit values. The assay was performed in triplicate and *T*_m_ values expressed as mean ± SD.

**Table 2:** *N*-glycan composition of gCTB expressed in *N. benthamiana*

Structure^a^		HPLC fraction^b^	Relative amount (%)^c^	
Oligomannosidic structure	Man_7_GlcNAc_2_	4-a	0.6	3.5
	Man_8_GlcNAc_2_	2	1.3	
	Man_9_GlcNAc_2_	4-b	1.6	
fucose/xylose-linked structure	Man_3_Xyl_1_GlcNAc_2_	10-a	14.7	
GlcNAc-linked structure	GlcNAc_1_Man_3_GlcNAc_2_^d^	9/17	6.2/33.1	
	GlcNAc_2_Man_3_GlcNAc_2_	15	12.5	51.8
Complex structure	GlcNAc_1_Man_3_Xyl_1_GlcNAc_2_^d^	10-b/16	3.3/16.7	
	GlcNAc_1_Man_3_Fuc_1_Xyl_1_GlcNAc_2_	3	0.8	24
	GlcNAc_2_Man_3_Xyl_1_GlcNAc_2_	14-a	3.2	
β1,3-Gal-linked structure	Gal_1_GlcNAc_2_Man_3_GlcNAc_2_	14-b	3.5	6.0
	Gal_1_GlcNAc_2_Man_3_Xyl_1_GlcNAc_2_	14-c	2.5	

^a^Man, mannose; GlcNAc, N-acetylglucosamine; Fuc, fucose; Xyl, xylose; and Gal, β1,3-galactose.

^b^Fraction numbers are shown in Fig. 5 and Fig. S2.

^c^The relative amount of each glycan was calculated from the fluorescence intensity of PA fractions in SF-HPLC.

^d^Two isomers for the terminal GlcNAc residue were identified.
