# Supplementary material for: N-Glycosylation of cholera toxin B subunit in Nicotiana benthamiana: impacts on host stress response, production yield and vaccine potential
Source: Sci Rep. 2015 Jan 23;5:8003. doi: 10.1038/srep08003 (PMC4303877; doi:10.1038/srep08003)
Supplement: Supplementary Information — Supplementary figures and legends [file srep08003-s1.pdf]

Title: *N*-Glycosylation of cholera toxin B subunit in *Nicotiana benthamiana*: impacts on host stress response, production yield and vaccine potential

Authors: Krystal Teasley Hamorsky[1,2], J. Calvin Kouokam[1,3], Jessica M. Jurkiewicz[1], Bailey Nelson[1], Lauren J. Moore[1], Adam S. Husk[1], Hiroyuki Kajiura[4], Kazuhito Fujiyama[4], and Nobuyuki Matoba[1, 3,*]

Affiliations: [1]Owensboro Cancer Research Program of James Graham Brown Cancer Center at University of Louisville School of Medicine, Owensboro, KY, USA

[2]Department of Medicine, University of Louisville School of Medicine, Louisville, KY, USA

[3]Department of Pharmacology and Toxicology, University of Louisville School of Medicine, Louisville, KY, USA

[4]The International Center for Biotechnology, Osaka University, Suita, Osaka, Japan

*To whom correspondence should be addressed: Nobuyuki Matoba, Owensboro Cancer Research Program of James Graham Brown Cancer Center, Department of Pharmacology and Toxicology, University of Louisville School of Medicine, 1020 Breckenridge Street Suite 201, Owensboro, KY 42303, USA, Tel: (270) 691 5955; Fax: (270) 685 5684; E-Mail: n.matoba@louisville.edu

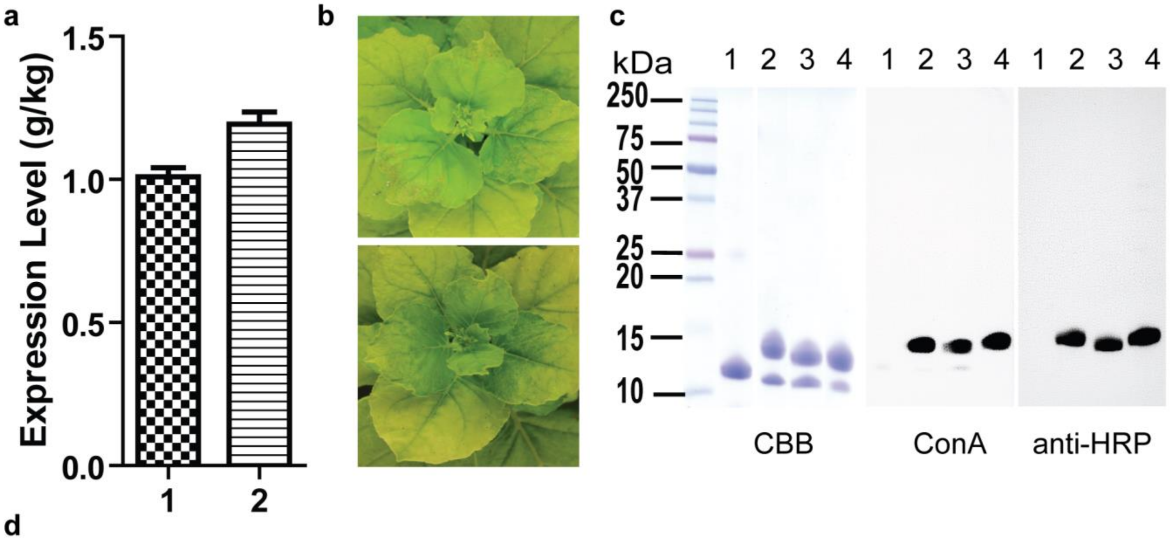

d

CTB:

TPQNITDLCAEYHNTQIHTLNDKIFSYTESLAGKREMAIITFKNGATFQVEVPGSQ
HIDSQKKAIERMKDTLRIAYLTEAKVEKLCVWNNKTPHAIAAISMAN

pCTB:

TPQSITDLCAEYHNTQIHTLNDKIFSYTESLAGKREMAIITFKNGATFQVEVPGSQ
HIDSQKKAIERMKDTLRIAYLTEAKVEKLCVWNNKTPHAIAAISMANSEKDEL

pCTB$^{\Delta KDEL}$:

TPQSITDLCAEYHNTQIHTLNDKIFSYTESLAGKREMAIITFKNGATFQVEVPGSQ
HIDSQKKAIERMKDTLRIAYLTEAKVEKLCVWNNKTPHAIAAISMAN

pCTB$^{\Delta KDEL\text{-}K23T}$:

TPQSITDLCAEYHNTQIHTLNDTIFSYTESLAGKREMAIITFKNGATFQVEVPGSQ
HIDSQKKAIERMKDTLRIAYLTEAKVEKLCVWNNKTPHAIAAISMAN

pCTB$^{\Delta KDEL\text{-}VTKALL}$:

TPQSITDLCAEYHNTQIHTLNDKIFSYTESLAGKREMAIITFKNGATFQVEVPGSQ
HIDSQKKAIERMKDTLRIAYLTEAKVEKLCVWNNKTPHAIAAISMANVTKAAL

Fig. S1. Analysis of the position of *N*-glycosylation on CTB. a Quantification of glycosylated CTB (1 - N4S-CTB-K23T; 2 – N4S-CTB-VTKALL) accumulation in leaf extracts at 5 dpi. GM1-ELISA was employed to quantify the amounts of receptor-binding CTB. Data are expressed as means ± SEM (n=3). Regardless of the glycosylation position, glycosylated CTB was accumulated higher than N4S-CTB (see Fig. 1). **b** Photographs showing the phenotype of N4S-CTB-K23T-expressing (Top) and N4S-CTB-VTKALL-expressing (Bottom) plants at 5 dpi. For both constructs there was little necrosis as with gCTB (see Fig. 1). **c** Detection of glycans in immunoblots using ConA and anti-HRP antibodies. Purified N4S-CTB-KDEL (lane 1), gCTB (lane 2), N4S-CTB-K23T (lane 3) and N4S-CTB-VTKALL (lane 4) were resolved by SDS-PAGE under denaturing conditions and then stained with Coomassie Brilliant Blue (CBB) or analyzed by immunoblotting using ConA and anti-HRP antibodies. ConA and anti-HRP antibodies both bound to gCTB, N4S-CTB-K23T and N4S-CTB-VTKALL, demonstrating the presence of plant *N*-glycans. In CBB stain, these glycosylated CTB variants showed a minor band at a lower position, which was not detected by ConA or anti-HRP antibodies and thus represents a non-glycosylated subunit. **d** Amino acid sequence of CTB variants. Underlined amino acids denote glycosylation sites; Red amino acids denote mutations.

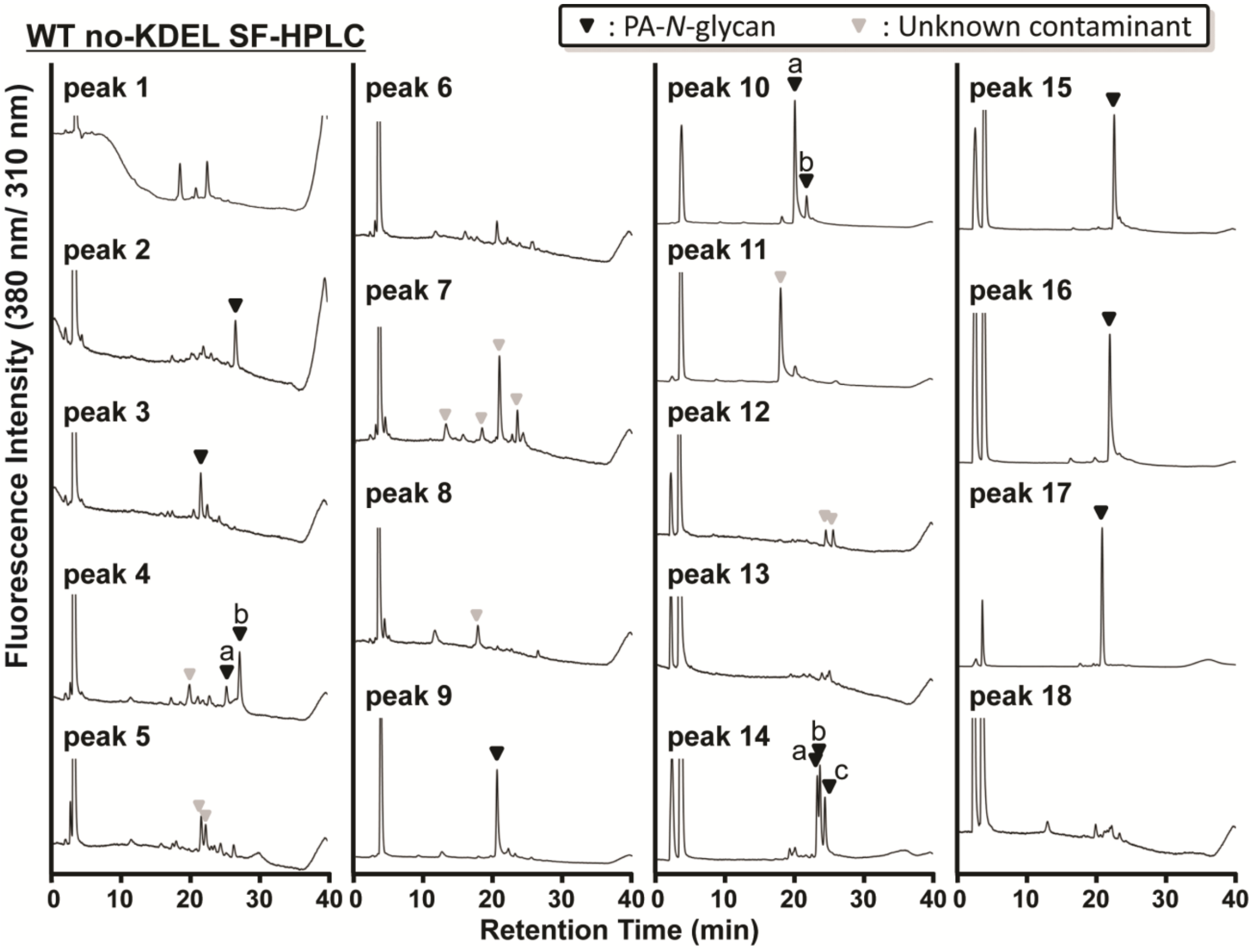

Fig. S2. SF-HPLC-based secondary separation of PA-glycans isolated from gCTB. The PA-labeled glycans (▼) separated by the initial RP-HPLC (Fig. 5) were further fractionated by SF-HPLC. The peak number shown in each chromatogram corresponds to that of RP-HPLC in Fig. 5. Lower case letters in chromatograms represent fractions subsequently analyzed for glycan mass and structure, as illustrated in Fig. S3.

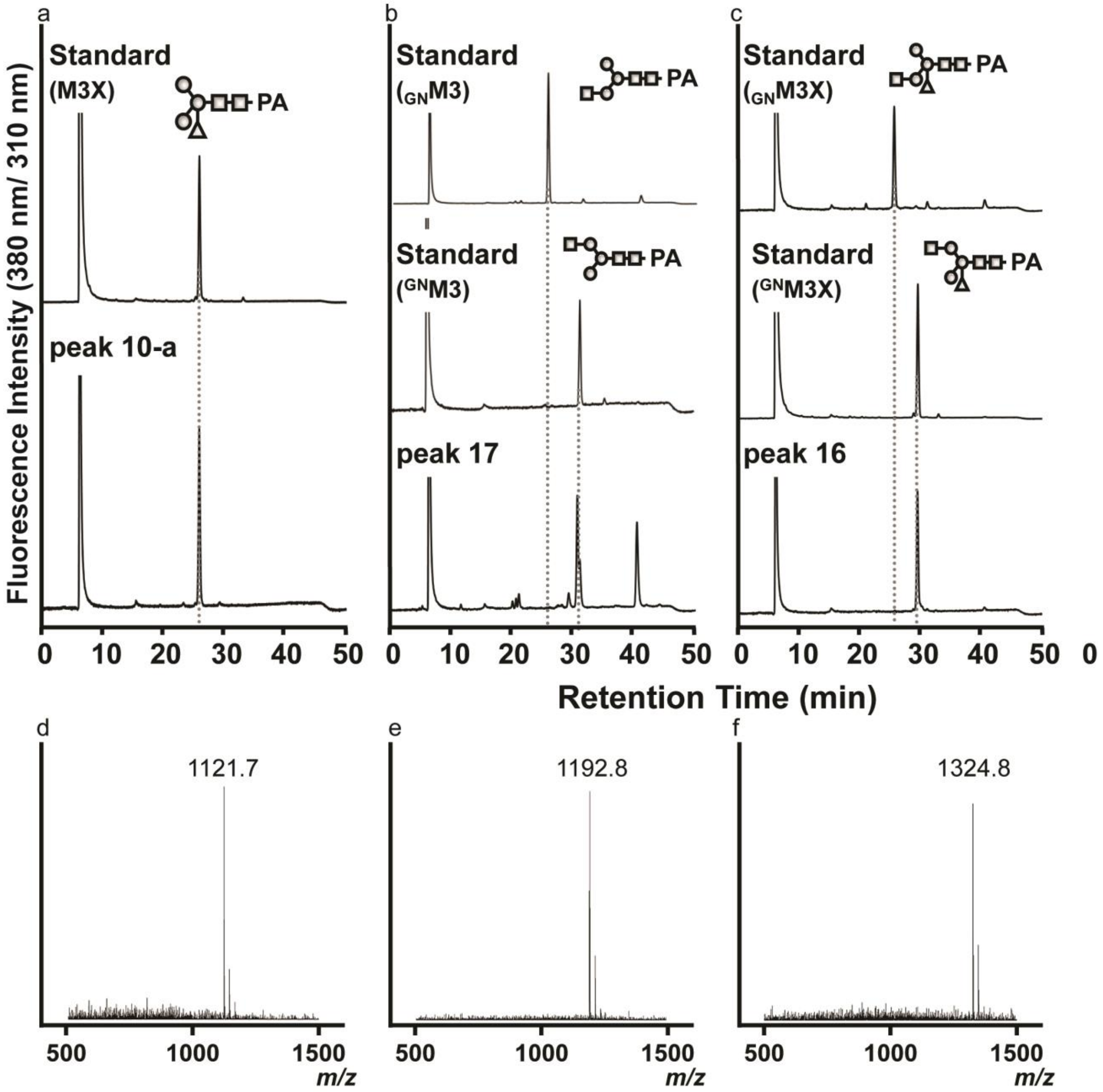

Fig. S3. Structural determination of representative PA-glycans isolated from gCTB. Detailed analysis for the three most abundant PA-glycan peaks, i.e., Peak 10-a (**a** and **d**), 17 (**b** and **e**) and 16 (**c** and **f**) are shown. Peak numbers correspond to those of SF-HPLC in Fig. S2. a-c, comparative RP-HPLC chromatograms showing the elution positions of peaks 10-a, 17 and 16 matching those of standard PA-labeled $Man_3Xyl_1GlcNAc_2$-PA (M3X), $GlcNAc_1Man_3GlcNAc_2$-PA (^{GN}M3) and $GlcNAc_1Man_3Xyl_1GlcNAc_2$-PA (^{GN}M3X), respectively. Note that two possible isomeric forms of $GlcNAc_1Man_3GlcNAc_2$-PA and $GlcNAc_1Man_3Xyl_1GlcNAc_2$-PA were analyzed in B and C, respectively. D-F, MALDI-TOF-MS analysis showing that the molecular masses of the subjects correspond to the theoretical values of RP-HPLC-determined glycan structures.

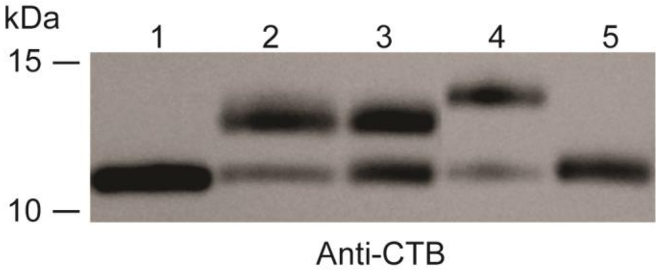

Fig. S4. Kif-gCTB is glycosylated with high-mannose-type glycans. For kifunensine treatment, immediately after gCTB infiltration, plants were removed from soil and submerged in water containing 5 μM kifunensine (Cayman Chemical, Ann Arbor, MI). On 2 and 4 dpi, fresh water containing 5 μM and 2.5 μM kifunensine, respectively, was added. On 5 days post infection, Kif-gCTB proteins were extracted and purified as described in the Materials and Methods. Endoglycosidase H (Endo H) cleaves *N*-linked high-mannose-type and hybrid (but not complex) glycans within the chitobiose core. CTB proteins (2 μg) were incubated with Endo H (2000 units) overnight at 37 ^{o}C, separated by SDS-PAGE, transferred to a PVDF membrane, and probed with anti-CTB antibodies. Kif-gCTB but not gCTB was completely cleaved by Endo H verifying the presence of high-mannose-type glycans. Numbers 1-5 correspond to N4S-CTB-KDEL, gCTB, gCTB + Endo H, Kif-gCTB, Kif-gCTB + Endo H, respectively.

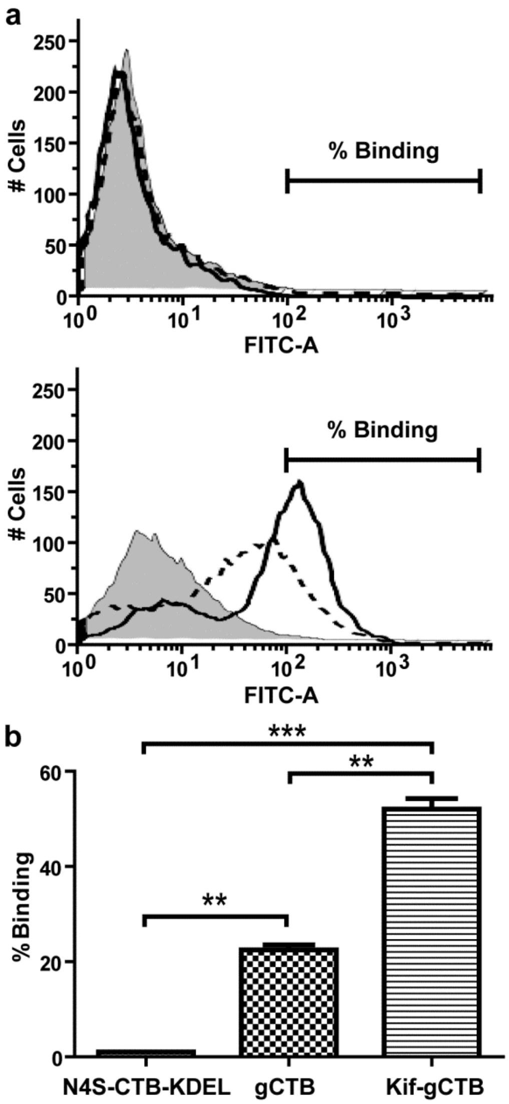

Fig. S5. gCTB binding to Dendritic cell-specific ICAM-3 grabbing nonintegrin (DC-SIGN). Flow cytometry analysis. N4S-CTB-KDEL, gCTB and Kif-gCTB were labeled with Alexa Fluor ® 488 (Molecular Probes) according to the manufacturer's protocol. Labeled N4S-CTB-KDEL, gCTB and Kif-gCTB at 20 µg/ml were pre-incubated with GM1-ganglioside (10 µM) overnight at 4°C to prevent protein binding due to the glycosphingolipid receptor on the cell surface. DC-SIGN expressed on the surface of Raji cells ($1x10^{6}$ cells; obtained from NIH AIDS Reagent Program) were incubated for 1h at room temperature while shaking at 150 RPM with pre-incubated N4S-CTB-KDEL, gCTB and Kif-gCTB, each at a final concentration of 10 µg/ml. Samples were washed two times in DPBS (GIBCO) by adding 200µL DPBS followed by centrifugation at 1000 RPM for 5 minutes at 4°C. Cell pellets were fixed in 200µL 1% paraformaldehyde and analyzed on a flow cytometer (BD FACSAria). As a control, Raji cells ($1x10^{6}$ cells) not expressing DC-SIGN treated in the same manner with labeled proteins were used to gate the cell population. **a** Representative histograms depicting binding of N4S-CTB-KDEL (shaded), gCTB (dashed line) and Kif-gCTB (solid line) binding to Raji (top) and Raji-DC-SIGN (bottom) cells. **b** Bar graph: The % binding of N4S-CTB-KDEL, gCTB and Kif-gCTB to DC-SIGN Raji cells. Data are expressed as means ± SEM (n=2). $^{**}P < 0.01$, $^{***}P < 0.001$ (one-way ANOVA with Bonferroni's multiple comparison test).

Table S1. Primers and amplicon characteristics for RT-qPCR.

Gene	Gene Name	Accession Number	RT-qPCR Primer Sequences Forward (F) and Reverse (R)	L	A	E (%)	R^2	Slope	y-int
BiP[a]	Binding immunoglobulin protein	X60057	**F** 5'-AGC TTT GAG CAG TCA ACA CCA AGT-3'<br>**R** 5'-AAA ACG TGC CCG AGT AAG TGG TTC-3'	992	91	107	0.976	-3.164	24.68
bZIP60[a]	Basic region leucine zipper motif 60	AB281271	**F** 5'-CCT GCT TTG GTT CAT GGG CAT CAT-3'<br>**R** 5'-AGA AGA CCG TGG TTT CTG CTT CGT-3'	927	99	98.1	0.987	-3.369	26.31
PDI[a]	Protein disulfide isomerase	Y11209	**F** 5'-TCC AAA GGG ATC ACT GGA GCC AAA-3'<br>**R** 5'-TCT GGA GAT AGC ACC ACA ACG CTT-3'	367	138	99.5	0.989	-3.334	25.46
SKP1[a]	S-phase kinase-associated protein 1	AF494084	**F** 5'-TGA CAT GCC AGA CAG TTG CAG ACA-3'<br>**R** 5'-AGG CAT TCT CCC TCC TGA CTT CTT-3'	326	123	105	0.996	-3.207	23.63
26Sα[b]	26S proteasome subunit alpha	DQ226996	**F** 5'-CAA CAG GGA GAA ACT CCA ACT C-3'<br>**R** 5'-CTG CCT CAA TCT CAG CAA CA-3'	146	216	108.7	0.981	-3.129	23.87
PR1a[b]	Pathogenesis-related protein 1a	X06930	**F** 5'-CCG TTG AGA TGT GGG TCA AT-3'<br>**R** 5'-CGC CAA ACC ACC TGA GTA TAG-3'	663	100	110.4	0.933	-3.095	29.85
18S[c]	18S ribosomal RNA	Y08501	**F** 5'-GCA AGA CCA AAA CTC AAA GG-3'<br>**R** 5'-TGT TCA TAT GTC AAG GGC TGG-3'	362375	107	100.9	1.000	-3.300	16.35

List of genes used in RT-qPCR. The designed primer sequences, location of amplicon (L) and amplicon length (A). Validation of qPCR shown by efficiency (E), R^2, slope and y-intercept (y-int) of the calibration curve. RT-qPCR reactions were set up in TempPlate semi-skirted 96-well PCR plates, natural (USA Scientific, Ocala, Fl) and sealed with TempPlate RT optically clear film (USA Scientific). For each plate, control amplifications using dilutions of non-infiltrated cDNA were used to determine efficiencies. Reactions were incubated at 95°C for 10 min, then for 40 cycles of 95°C for 15 s, followed by 65°C for all primer sets except *PR1a* and *26Sα*, for which 50°C and 60°C were used, respectively, for 60 s. Duplicate reactions were carried out for each sample and averaged. The calibration curve was obtained from the iQ5 Optical System Software Version 2.1. [a]Primers obtained from (Ye et al., 2011). [b]Primers were designed using PrimerQuest by Integrated DNA Technologies (https://www.idtdna.com/Primerquest/Home/Index). [c]Primers obtained from (Liu et al., 2012).

Liu, D., Shi, L., Han, C., Yu, J., Li, D. and Zhang, Y. (2012) Validation of reference genes for gene expression studies in virus-infected Nicotiana benthamiana using quantitative real-time PCR. *PloS one* **7**, e46451.

Ye, C., Dickman, M.B., Whitham, S.A., Payton, M. and Verchot, J. (2011) The unfolded protein response is triggered by a plant viral movement protein. *Plant Physiol* **156**, 741-755.

**Gene**	***18S***	***PP2A***	***F-Box***
Number of Samples	24	24	24
Geometric Mean [C_q]	**18.13**	**25.52**	**27.44**
Arithmetic Mean [C_q]	**18.14**	**25.53**	**27.45**
Min [C_q]	17.24	24.96	26.72
Max [C_q]	19.18	27.93	28.71
Standard Deviation [± C_q]	**0.32**	**0.40**	**0.40**
Coefficient of Variation [% C_q]	**1.78**	**1.57**	**1.44**
Coefficient of Correlation [r]	**0.861**	**0.684**	**0.606**
p-value	**0.001**	**0.001**	**0.002**

Table S2. BestKeeper results of the three candidate reference genes based on their quantification cycle (C_q). The standard deviation, coefficient of variation and coefficient of correlation were all used to evaluate the stability of the candidate reference genes. The data shows that *18S* is the most stable reference gene at a significant level ($p < 0.001$).
